# AKR1B10 and digestive tumors development: a review

**DOI:** 10.3389/fimmu.2024.1462174

**Published:** 2024-12-16

**Authors:** Yao Shen, Ailin Qiu, Xin Huang, Xiaosha Wen, Sundar Shehzadi, Yan He, Qian Hu, Jian Zhang, Dixian Luo, Shenghui Yang

**Affiliations:** ^1^ Medical School, Hunan University of Chinese Medicine, Changsha, Hunan, China; ^2^ Institute of Pharmacy and Pharmacology, School of Pharmaceutical Science, Hengyang Medical School, University of South China, Hengyang, Hunan, China; ^3^ Laboratory Medicine Center, Shenzhen Luohu Hospital Group, the Third Affiliated Hospital (The Affiliated Luohu Hospital) of Shenzhen University, Shenzhen University, Shenzhen, Guangdong, China; ^4^ First Affiliated Hospital of Anhui Medical University, Hefei, Anhui, China; ^5^ Department of Preventive Medicine, Medical School, Hunan University of Chinese Medicine, Changsha, Hunan, China

**Keywords:** AKR1B10, metabolism, mechanisms, review, digestive system tumors

## Abstract

Aldo-keto reductase family 1 member B10 (AKR1B10) is a member of the AKR1B subfamily. It is mainly found in cytoplasm, and it is typically expressed in the stomach and intestines. Given that its expression is low or absent in other tissues, AKR1B10 is a potential diagnostic and therapeutic biomarker for various digestive system diseases. Here, we review recent research progress on AKR1B10 in digestive system tumors such as hepatocellular carcinoma, gastric carcinoma, colorectal carcinoma, pancreatic carcinoma, oral squamous cell carcinoma, laryngeal squamous cell carcinoma, cholangiocarcinoma, and nasopharyngeal carcinoma, over the last 5 years. We also discuss the current trends and future research directions for AKR1B10 in both oncological and non-oncological diseases to provide a scientific reference for further exploration of this gene.

## 1 Introduction

AKR1B10 is an NADPH-dependent reductase belonging to subfamily 1B of the aldo-keto reductases (AKRs). It was first discovered by Professor De-Liang Cao in 1998 (1). AKR1B10 encodes proteins that catalyze the reduction of aldehydes, ketones, and quinones. It interacts with heat- shock protein 90α and regulates lipid synthesis via acetyl coenzyme A carboxylase α (ACCα), thereby playing a central role in cancer lipid metabolism (2).

According to RNA-sequencing and immunohistochemistry (IHC) analysis, the enzyme is highly expressed in the stomach, small intestine, and colon, but its expression is downregulated in gastrointestinal (GI) cancers and inflammatory bowel disease (3–7). At the same time, it is increased in normal tissues such as the liver, thymus, and prostate. Its expression is upregulated in the presence of cancer, nonalcoholic fatty liver disease (NAFLD), and certain skin diseases (8–11). Further, the expression of this enzyme is elevated in cancer cells that are resistant to clinical anticancer drugs such as ethoxyquin, doxorubicin, and lipopolysaccharide (12–14), suggesting a potential association between the AKR1B10 expression and resistance to chemotherapy. This underscores the growing evidence that AKR1B10 could be a promising target for the diagnosis and treatment of tumors and other related diseases (11).

In this paper, we review the research progress on AKR1B10 in digestive system tumors in the last 5 years, and provide a reference for the subsequent research of AKR1B10 in more diseases.

## 2 Structure and function of AKR1B10

### 2.1 AKR1B10 structure

Based on sequence similarity, the AKR family is divided into 16 major classes, namely, AKR1-AKR16, with each number representing a different class, and each family is further divided into subfamilies based on >60% sequence homology with each other. There have been 15 AKR family members identified thus far, including AKR1A, AKR1B, AKR1C, AKR1E, AKR6A, and AKR7A subfamilies. Among them, AKR1B1, AKR1B10, and AKR1B15 in the AKR1B subfamily have been widely studied for their role in tumors (11–15). AKR1B10 shares more than 68% homology with AKR1B1 and up to 91.5% homology with AKR1B15 (1, 16, 17), so AKR1B10 is often compared to both in studies of enzymatic function and inhibitors.

**Figure 1:**
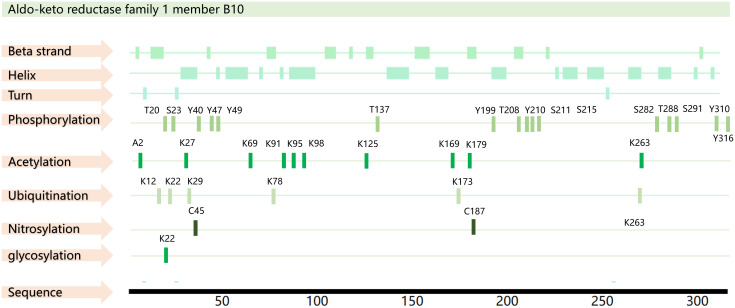
AKR1B10 sequence and PTM sites. The amino acid sequence of AKR1B10 was obtained from the UniProt database (https://www.uniprot.org/), and the PTM sites were obtained from the PhosphoSitePlus database(https://www.phosphosite.org/) and PTMcode 2 database(PTMcode 2: Home (embl.de)).

Structurally, AKR1B10 contains beta strands, alpha helices, and turn features, as well as a macrocycle that controls substrate specificity and a conserved cofactor- binding domain. A total of 35 posttranslational modifications have been predicted for AKR1B10, including phosphorylation modifications at 16 sites, acetylation modifications at 10 sites, ubiquitination modifications at 6 sites, nitrosylation modifications at 2 sites, and glycosylation modification at 1 site (18–20) (**Figure 1**). However, further exploration is needed to determine the actual function of these modification sites.

### 2.2 AKR1B10 function

AKR1B10, also known as AKR1B11, exhibits catalytic activity for all-trans retinaldehyde, H+, and NADPH (21, 22). It facilitates the reduction of aldehydes and ketones carboxylic compounds, thereby mitigating damage to proteins and DNA. This detoxification helps to maintain cellular homeostasis and thereby protects the cells. Its potent retinaldehyde reductase activity can indirectly influence cell differentiation (23). This enzyme also interacts with ACCα to inhibit apoptosis by upregulating ACCα expression to promote lipid synthesis, reduce mitochondrial membrane damage, and inhibit cytochrome c release and caspase-3 activation (24, 25).

## 3 AKR1B10 and tumors of the digestive system

The development of tumors necessitates a restructuring of cellular metabolism, which underscores the significance of understanding the role of metabolism in tumorigenesis. Metabolic alterations are a notable hallmark of tumors and can profoundly affect various functions of both normal and cancerous cells, including cell proliferation and migration. Progression of gastrointestinal (GI) tumors is closely related to metabolism; thus, metabolomics studies offer fresh perspectives on the metabolic mechanisms of GI tumors. The principal metabolic changes associated with tumors encompass abnormal uptake of glucose and amino acids, and the generation of essential substances and NADPH through metabolic pathways (26). Functioning as an NADPH-dependent reductase, AKR1B10 assumes a critical role in metabolic activities, such as lipid synthesis, transport, oxidation, drug metabolism, and cellular signaling. Further, AKR1B10 stands as a key participant in the cellular antioxidant defense mechanism, essential for maintaining intracellular redox homeostasis via reduction reactions (11, 25).

**Figure 2:**
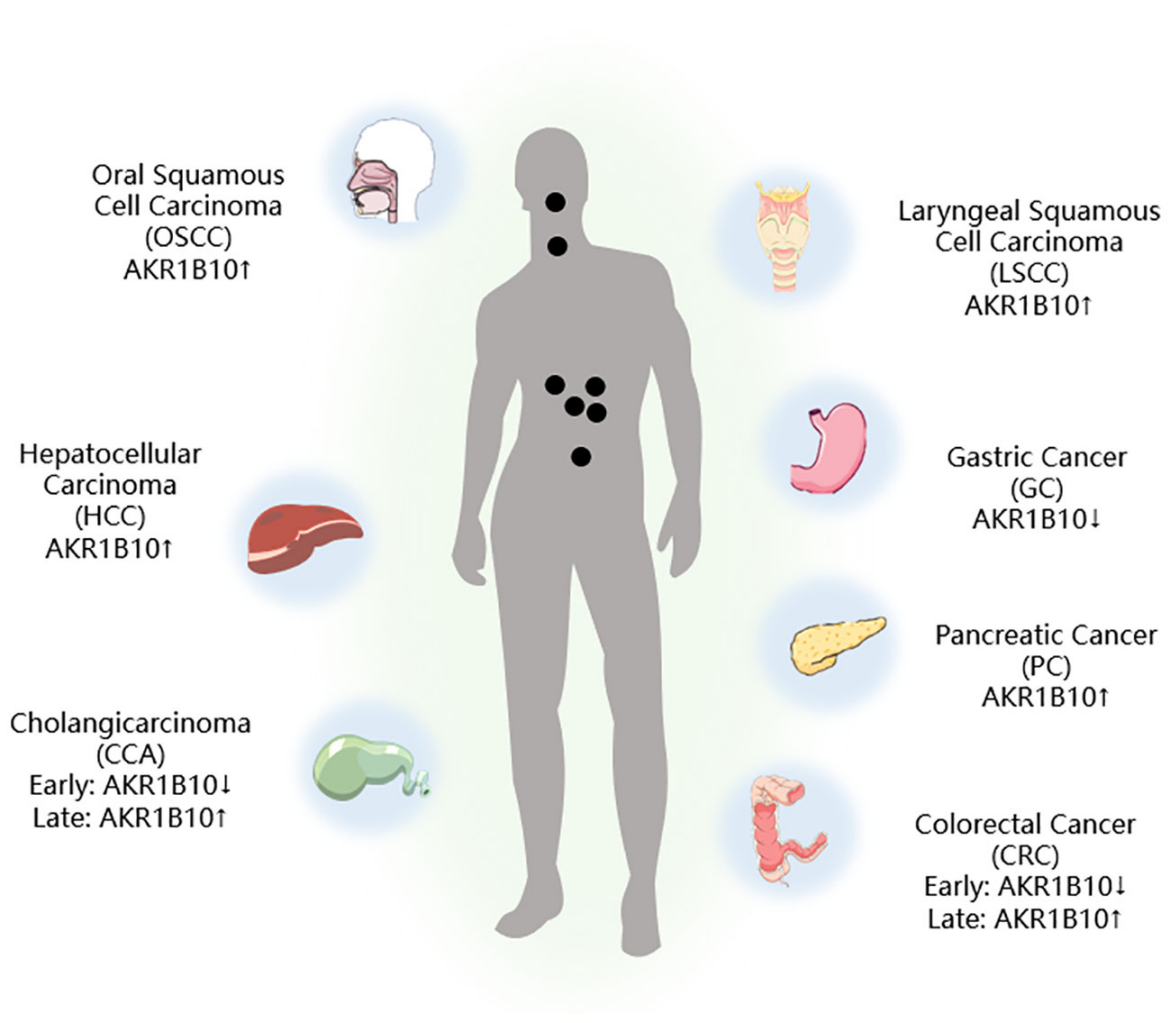
AKR1B10 expression in digestive tract tumors. ↑: Upregulation of AKR1B10 expression; ↓: Downregulation of AKR1B10 expression.

The mRNA and protein levels of 12 known AKR family members, namely, AKR1A1, AKR1B1, AKR1B10, AKR1B15, AKR1C1, AKR1C2, AKR1C3, AKR1C4, AKR1D1, AKR1E2, AKR7A2, and AKR7A3, have been evaluated by next-generation sequencing (NGS) and liquid chromatography-tandem mass spectrometry (LC-MS/MS). Most of the AKR isoforms were found to be highly expressed in the duodenum and jejunum regions, and the expression declined toward the rectum, with AKR1B10 having the highest expression level (27–29). Consequently, conducting thorough research into the connection between AKR1B10 and GI tumors holds significant promise (**Figure 2**).

### 3.1 AKR1B10 and liver cancer

Lipids are mainly processed and metabolized in the liver. Under normal conditions, the liver can break down excess cholesterol, triacylglycerols, and glycerophospholipids, and re-release them into the blood circulation. Liver processes that act on lipids include fatty acid synthesis (FAS) and fatty acid oxidation (FAO). When the liver function is affected by the environment, drugs, genetics, and diseases and cannot synthesize and metabolize lipids properly, excess lipids accumulate in the liver, affecting its function and gradually forming fatty liver, then cirrhosis, and ultimately developing into hepatocellular carcinoma (HCC) (30–33).

HCC is the most prevalent form of liver cancer. It is known for its aggressive nature, and ranks among the top three causes of cancer-related deaths globally (34). Despite advancements in diagnostic techniques and surgical treatments, the 5-year survival rate for patients with advanced HCC remains low, which is largely a consequence of the challenge of early detection (35).

It has been demonstrated that HCC can evolve from NAFLD, and AKR1B10 expression is upregulated in NAFLD, which is consistent with AKRB10 expression in HCC cells (9). In HCC, changes in lipid metabolic processes, such as FAS and FAO, can affect cancer cell proliferation compared with normal tissues. Specifically, upregulation of FAS may provide cancer cells with lipids required for the construction of new cell membranes, while an increase in FAO may provide additional energy to support rapid proliferation of cancer cells. Consequently, inhibiting the FAS and FAO processes may reduce the energy supply to HCC cells, and limit their growth (36). Blocking AKR1B10 expression leads to cell cycle arrest and impaired cell proliferation, indicating a potential tumorigenic role for AKR1B10 in promoting cell growth (37).

The results of a meta-analysis have shown that high expression of AKR1B10 predicts a favorable prognosis after hepatectomy (38). AKR1B10 has an overall sensitivity and specificity of 78% and 85%, respectively, for diagnosing HCC, and it appears more sensitive than alpha-fetoprotein (AFP) for detecting early-stage HCC. Further, combining AKR1B10 and AFP shows higher sensitivity and specificity for HCC diagnosis compared with using AKR1B10 or AFP alone (38). However, in the current state of research, AKR1B10 has not been widely used as a predictive test for HCC compared to AFP in clinical applications, and further comprehensive studies or large randomized controlled multicenter trials are required to delve deeper into the clinical significance of AKR1B10 in patients with HCC.

The researchers used the DepMap dataset to analyze AKR1B10 by gene effector CRISPR (DepMap Public 22Q4 + Score, Chronos) and found that upregulation of AKR1B10 expression is associated with poorer overall survival, and that HCC cell proliferation, migration, and invasion are influenced by AKR1B10 activity (39). Mechanistically, AKR1B10 increases the expression of cell proliferation and the EMT-associated proteins CCND1, E-cadherin, N-cadherin, vimentin, and Twist1. AKR1B10 knockdown results in decreased levels of PI3K and AKT phosphorylation, suggesting that AKR1B10 promotes proliferation, migration, and invasion of HCC cell through the PI3K/AKT signaling pathway, and this pathway has an important reference value for the assessment of HCC (39–43).

According to Liu et al., during the progression of HCC, AKR1B10 may be expressed as a compensatory mechanism to protect hepatocytes from oxidative stress (44). This upregulation could be a response to chronic liver disease and hepatocarcinogenesis, while the absence of AKR1B10 might accelerate hepatotoxin and inflammation-related hepatocarcinogenesis. Therefore, rather than being a driver of malignant transformation during HCC development, increased AKR1B10 expression in HCC may be a compensatory mechanism.

### 3.2 AKR1B10 and gastric cancer

Gastric cancer (GC) is a widespread malignant tumor with a grim prognosis (45). The expression of AKR1B10 in GC tissues is markedly lower than that in normal gastric tissues. In addition, clinicopathological factors suggest that increased AKR1B10 expression predicts a poor prognosis in patients with GC undergoing resection (46).

The proliferation and spread of GC cells are driven by the abnormal activation of epithelial-mesenchymal transition (EMT), and EMT can be activated by factors such as TGF-β and mesenchymal markers (Slug, vimentin, and α-SMA), which promote cancer development. Selective inhibition of heparinase with suramin can inversely inhibit EMT activation and thereby retard the proliferation and migration of GC cells (47, 48). Notably, in early GC, all of the water-soluble compounds and volatile metabolites explored were found to be lipids, hinting at a close tie between abnormal lipid metabolism and GC development (49). Furthermore, AKR1B10 plays a role in lipid synthesis, cellular metabolism, and fatty acid oxidation. Investigating the link between AKR1B10 and GC is an intriguing area of study (25). It has been confirmed that AKR1B10 exerts a regulatory influence on EMT, which is inversely linked to with tumor volume, infiltration depth, and metastasis. Moreover, its positivity serves as a predictor of a better 5-year survival rate for patients with GC (50).

However, in contrast to the above results, Ahmed et al. verified that AKR1B10 was expressed predominantly in the cytoplasm of GC cells and that positive expression of AKR1B10 was associated with lymph node metastasis and poorer tumor response to neoadjuvant chemotherapy, which indicated a poorer prognosis for the patient (7, 46). A meta-analysis has shown that AKR1B10 expression is actually not associated with overall patient survival (51). These differences may be related to a number of factors, including the different reagent products, antibody brands, and sample sources used by investigators to validate *in vivo*, and *in vitro* experiments. Specifically, reagents from different manufacturers may vary in purity, activity, and stability, all of which may affect the consistency and reproducibility of experimental results. Similarly, antibodies from different brands may differ in specificity and affinity, which in turn may affect the accuracy of the experiment. In addition, the diversity of sample sources, such as genetic backgrounds, pathological states, and environmental exposures of different individuals, may also have a significant impact on experimental results. Therefore, to ensure the reliability and validity of experimental results, we suggest that these potential sources of variation should be fully considered in experimental design and data analysis, and appropriate measures should be taken to control and correct the effects of these variables.

Integrin subunit alpha 5 (ITGA5) is involved in cell surface adhesion and signaling. It is upregulated in GC tissues and cells. Salvage experiments have suggested that AKR1B10 may act as a potential tumor suppressor in GC, inhibiting the migration, invasion, and adhesion of GC cells by modulating ITGA5 expression (52). However, the regulatory mechanism of AKR1B10 on ITGA5, as well as how both affect the proliferation and migration of GC cells, remain unproven, and the available data are insufficient to support further studies.

The feasibility of targeting AKR1B10 is unclear due to the differences in its expression in GC and other tumors, as well as the discrepancies in findings between different investigators (35, 53). Few studies have confirmed the value of AKR1B10 in diagnosis, and prognosis prediction, and in-depth mechanistic studies are lacking. Therefore, it is important to further investigate whether AKR1B10 can be a potential target for GC treatment.

### 3.3 AKR1B10 and colorectal cancer

Colorectal cancer (CRC) is a prevalent and lethal form of cancer worldwide. Its intricate mechanisms hamper the effectiveness of treatments for advanced cases. Therefore, gaining insight into the mechanisms underlying CRC occurrence and progression is crucial for identifying new treatment targets. Previous studies have indicated that unusually low levels of AKR1B10 in the GI tract are closely associated with cancer development (4, 54). Yet, the role of AKR1B10 in CRC development remains poorly understood, making the exploration of its role and molecular mechanism in CRC an active area of research.

The gene AKR1B10 is typically found in intestinal epithelial cells, but its expression is low in the early stages of CRC and increases as the cancer progresses. Both *in vitro* and *in vivo* functional assays have shown that AKR1B10 expression is downregulated in CRC and is linked to the patients’ clinicopathological status (3). Deletion of AKR1B10 has been found to enhance the proliferation and migration of CRC cells *in vitro*, whereas overexpression of AKR1B10 has the opposite effect. In addition, patients with high AKR1B10 expression have been shown to have longer overall survival (6).

Fibroblast growth factor 1 (FGF1) plays a critical role in maintaining lipid and metabolic homeostasis, and it exhibits anti-inflammatory effects (55, 56). This suggests that FGF1 could potentially affect tumor progression through its influence on metabolic disorders. Further, AKR1B10 shows significant association with FGF1 gene and protein levels. *In vivo* experiments have shown that downregulation of AKR1B10 promotes tumor growth and increases FGF1 expression, suggesting that AKR1B10 may play a tumor-suppressive role in CRC by decreasing the level of FGF1 (3). *In vitro* studies have also demonstrated that ectopic expression of AKR1B10 significantly inhibits the proliferation, clone formation, and migration of CRC cells (54).

Liu et al. demonstrated for the first time that the expression of AKR1B10 significantly correlated with the TNM stage and clinical stage of human colon cancer. Moreover, they found that AKR1B10 promoted the production of IL-1α and IL-6 in colon cancer cells through the activation of NF-κB, and the proliferation of cancer cells was inhibited by knocking down AKR1B10, which contrasts some previous findings (57). This may be closely related to AKR1B10’s aldose reductase activity, whose protumorigenic effects are attenuated upon inhibition of the inflammatory factors IL-1α and IL-6, thereby inhibiting tumor cell growth. The latest study has shown that AKR1B10 was positive in only 12.16% of the tumor tissues of 592 patients with CRC, while AKR1B10 was not detected in 63.13% of tumor tissues, so it is speculated that AKR1B10 may be an oncogenic factor rather than a prognostic indicator for CRC (58).

In the examination of AKR1B10 in relation to autophagy, it has been discovered that when AKR1B10 interacts with GAPDH, it can trigger an NADPH-dependent reduction reaction, leading to the reduction of GAPDH. This prevents the translocation of GAPDH into the nucleus, thereby impeding autophagy progression during glucose deprivation. This study implies that not only does AKR1B10 hinders the nuclear translocation of GAPDH by interacting with it, but it also obstructs the conversion of normal cells to cancer cells by suppressing the downregulation of autophagy through AMPK phosphorylation (59). These findings offer valuable insights into the regulation of autophagy in human colon cancer.

Further, there is evidence suggesting that elevated levels of arachidonic acid (AA) can disrupt the gut microbial balance, thereby potentially contributing to the development of CRC. The drug inhibition assay revealed that AA had an inhibitory effect on AKR1B10 with an IC50 value of 1.1 μM, and was able to effectively inhibit AKR1B10-mediated 4-oxo-2-nonenal metabolism (60). In previous studies, CRC has usually been associated with inflammation and intestinal flora, which are also closely related to metabolic processes. There are no data to support whether AKR1B10 affects the development of CRC through changes in the inflammatory microenvironment or flora, suggesting that we can start from this direction in our future work to reveal the role of AKR1B10 in the inflammatory microenvironment and the intestinal flora of CRC.

### 3.4 AKR1B10 and pancreatic cancer

Risk factors for pancreatic cancer (PC) are closely associated with lifestyle, diet, environment, genetic factors, and genetic environmental interactions (61). Commonly recognized risk factors for PC development include smoking and chronic pancreatitis (62, 63). Excessive accumulation of nutrients and metabolites can also disrupt the body’s metabolic environment, potentially leading to direct carcinogenic effects (64, 65). Overexpression of AKR1B10 has been identified in smoking-related cancers, such as lung cancer. Since the development of PC is closely related to smoking, and AKR1B10 can be activated by tobacco-associated oncogenic transcription factors (66, 67), it has been hypothesized that AKR1B10 expression is upregulated in human PC, which was confirmed by immunohistochemical (IHC) results (68, 69).

Upon evaluating the expression and enzymatic activity of AKR1B10 in isolated human PC samples, it has been observed that AKR1B10 expression is notably increased in pancreatic precursor lesions and invasive adenocarcinomas. Knocking down AKR1B10 in PC cells results in the inhibition of the Kras and its downstream Kraf/MEK/ERK pathway, along with the upregulation of E-cadherin expression (70). This leads to the suppression of the proliferation, invasion, and metastasis of PC cells. In summary, the downregulation of AKR1B10 expression is linked to heightened apoptosis, decreased protein prenylation, and inhibited Kras and its downstream effectors activation. Targeting protein prenylation, including AKR1B10 and Kras and its downstream pathways, and inducing apoptosis hold substantial promise for future research (68).

Checkpoint Suppressor 1 (CHES1) is a member of the forehead box (Fox) family of proteins that inhibit PC proliferation and invasion by regulating cellular senescence. Proteomic analyses have shown that CHES1 inhibits AKR1B10 expression, thereby suppressing PC cell activity and senescence phenotype (71). This not only suggests the feasibility of inhibiting PC progression from the CHES1/AKR1B10 signaling pathway, but also provides new ideas for cellular senescence therapies.

### 3.5 AKR1B10 and oral cancer

There are various histological types of oral cancer, such as squamous cell carcinoma derived from epithelial cells, adenocarcinoma from salivary glands, lymphoma from tonsils, and melanoma from melanin-producing cells. Oral squamous cell carcinoma (OSCC) is the most prevalent tumor, accounting for approximately 90% of oral cancers (72). In Southeast Asia, OSCC is primarily linked to the use of tobacco, alcohol, and particularly betel nut (73).

There is a significant correlation between AKR1B10 and tumor size, perineural infiltration, and recurrence in OSCC (74). Elevated expression of AKR1B10 is associated with poor overall survival in OSCC, suggesting its potential as a prognostic marker (51). High levels of salivary AKR1B10 may also be linked to disease progression and poor prognosis in OSCC (75). Combining AKR1B10 immunostaining with clinicopathological features enables the categorization of patients into different risk groups, which could aid in better clinical management of OSCC and the identification of effective targeted therapies for AKR1B10-associated malignancies (74). In addition, metabolomics analysis has revealed potential disruptions in amino acid and lipid metabolism in patients with OSCC, emphasizing the link between OSCC and AKR1B10 (76). However, further research is needed to delve into the molecular mechanisms underlying the role of AKR1B10 in OSCC.

### 3.6 AKR1B10 and laryngeal cancer

Laryngeal squamous cell carcinoma (LSCC) originates from the epithelial tissue of the laryngeal mucosa and accounts for 90% all of laryngeal cancers (77, 78). Despite advancements in surgery and radiotherapy, the mortality rate of LSCC remains high. Consequently, there is a growing focus on exploring the molecular mechanisms involved in LSCC development and devising new therapeutic approaches.

Liu et al. observed high expression of AKR1B10 in LSCC, with its level of expression being inversely associated with differentiation and positively correlated with tumor size (79). Moreover, AKR1B10 has been found to be overexpressed in Hep-2 laryngeal carcinoma cells, and inhibiting its activity and expression in these cells with oleanolic acid resulted in inhibited proliferation, migration, and invasion (79). The microenvironment of LSCC is also influenced by glucose metabolism, lipid metabolism, and nitrogen metabolism (80–83). Thus, it is plausible that AKR1B10 expression is linked to LSCC development, and it stands as one of the potential prognostic indicators for the condition. However, the existing studies on AKR1B10 in LSCC are limited, and further studies with an ample number of samples are required to quantify AKR1B10 expression at the tissue level for a more comprehensive understanding of how AKR1B10 influences the phenotype of LSCC.

### 3.7 AKR1B10 and biliary cancer

Cholangiocarcinoma (CCA) is a relatively rare type of cancer within the digestive system, but it is the most common aggressive malignant tumor of the biliary tract. It is closely associated with metabolism and is the second most prevalent primary malignant tumor of the liver, following HCC. CCA mainly originates from the epithelium of the bile ducts and can affect the entire biliary tract (84, 85). Changes in amino acid and lipid metabolism during the development of CCA provide ample nutrients for the growth and spread of cancer cells (86). This underscores the close relationship between CCA and metabolism. Diagnosis of CCA is often delayed due to the lack of obvious clinical symptoms, resulting in many patients having reached advanced stages at the time of diagnosis. Limited understanding of the molecular mechanisms of CCA further restricts early diagnosis and treatment.

According to the IHC analysis, AKR1B10 expression is upregulated during the middle and early stages of high differentiation, and downregulated in the later stage of low differentiation in CCA (87). This indicates that AKR1B10 could serve as a valuable marker for CCA proliferation and differentiation. Cai et al. revealed an upregulation pattern of AKR1B10 expression and its oncogenic effects in CCA. Their investigation of genes associated with AKR1B10 led to the discovery that the tumor-promoting function of methyltransferase 3 (METTL3) relied on the N6-methyladenosine (m6A) modification of AKR1B10. Further, they found that the knockdown of AKR1B10 reversed the tumor-promoting effects induced by METTL3 overexpression (88). These findings open up new avenues for future studies on the role of AKR1B10 in tumors.

## 4 AKR1B10 inhibitors

The differential expression of AKR1B10 in tumor and normal tissues has prompted the exploration of its role in tumor therapy. The quest for effective AKR1B10 inhibitors has the potential to enhance the efficacy of chemotherapy and open up new prospects for clinical cancer treatment. These inhibitors can be broadly categorized into four groups, namely, aldose reductase inhibitors (ARIs), endogenous substances, and natural and chemically synthesized sources (89). Among natural inhibitors, frangula emodin, aloe emodin, frangulin A, and frangulin B have demonstrated superior inhibition of AKR1B10 compared with AKR1B1, with IC_50_ values falling within the low micromolar range (3.5-16.6 μM) (90). Oleanolic acid, a triterpenoid, stands as an earlier and widely used natural inhibitor, exhibiting higher selectivity for AKR1B10 compared with AKR1B1, potentially attributed to the nonconserved residues Val301 and Gln303 in AKR1B10 (91). In addition, the aldose reductase inhibitor epalrestat, despite its potential for causing DNA damage, when combined with drugs such as sorafenib and doxorubicin, enhances the sensitivity of cancer cells to the drug (92, 93). 7-Hydroxy-2-oxo-2H-chromene-3-carboxylic Acid [3 (4-Fluorophenyl) propyl] amide (HCCFA) demonstrates more stable inhibition among chemosynthetic inhibitors (94–96). Moreover, in Ejaz’s computer simulation study, two quinolones, namely, quinine and quinidine, have been anticipated to be potential AKR1B10 inhibitors (97). Overall, the prediction of small molecule drugs targeting AKR1B10 through network pharmacology, molecular docking, and molecular dynamics simulation stands as a promising approach for screening potential inhibitors. Experimental validation in tandem with the screening results will provide a reliable scientific reference for new drug development (**Table 1**).

**Table 1:** Some AKR1B10 inhibitors, and their drug half-inhibitory concentrations (IC_50_), and chemical structural formulas.

Drug	IC_50_(μM) AKR1B10 AKR1B1		Structure	References
Frangula emodin	3.47	>50	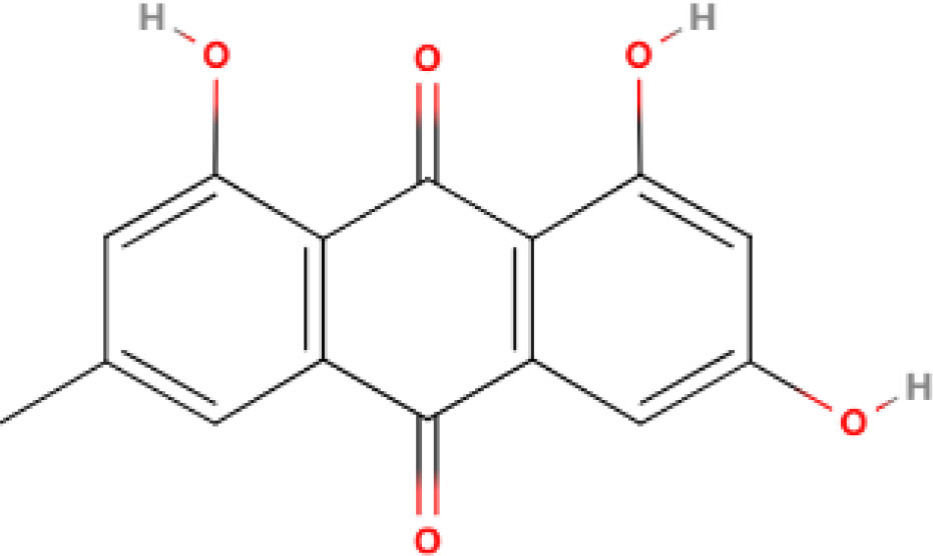	(90)
Aloe emodin	16.6	>50	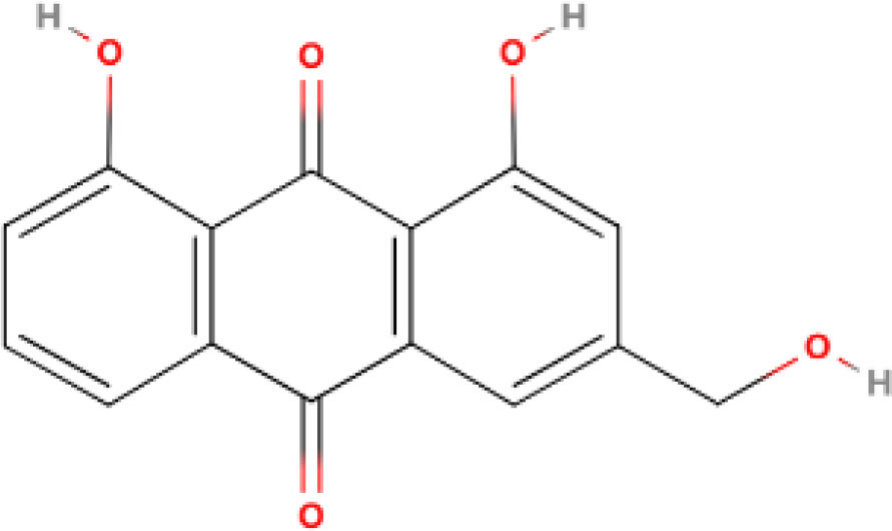	(90)
Frangulin A	5.73	>50	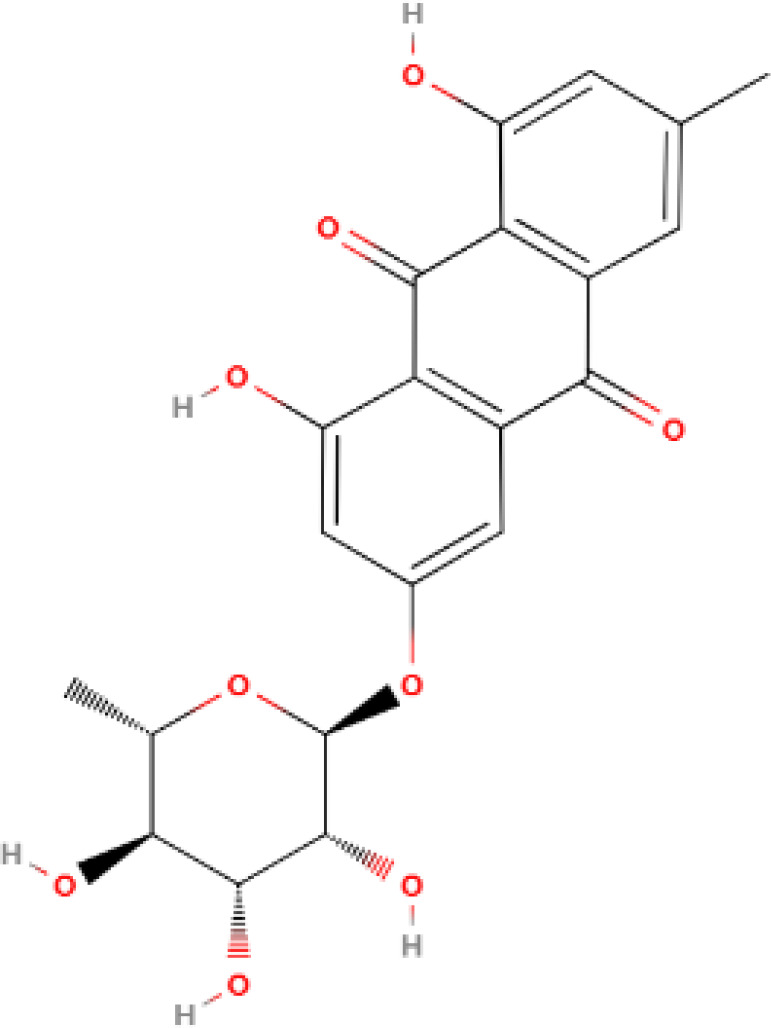	(90)
Frangulin B	5.45	>50	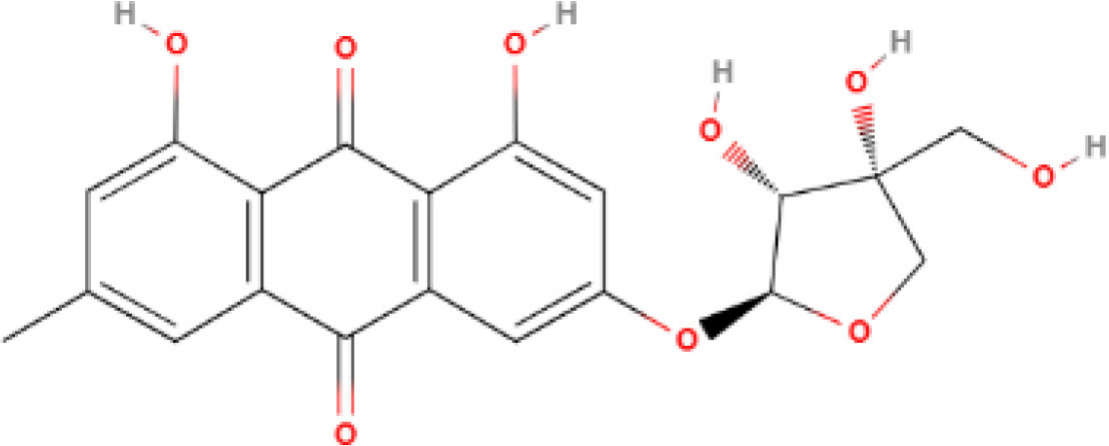	(90)
Oleanolic acid	0.090	124	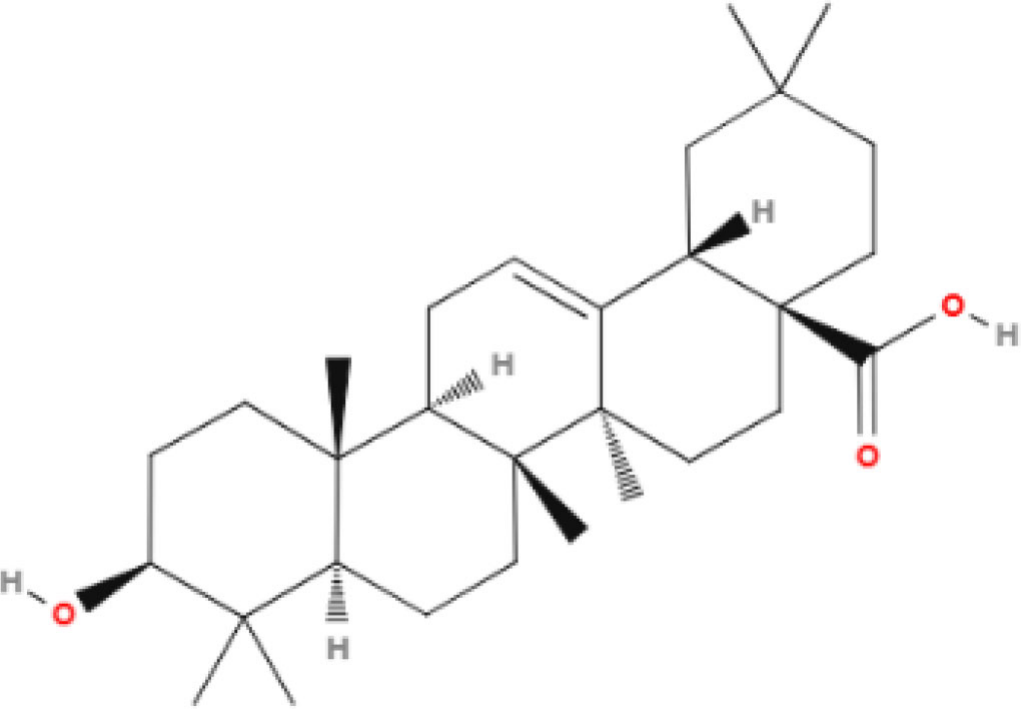	(11, 91)
Epalrestat	0.33	0.021	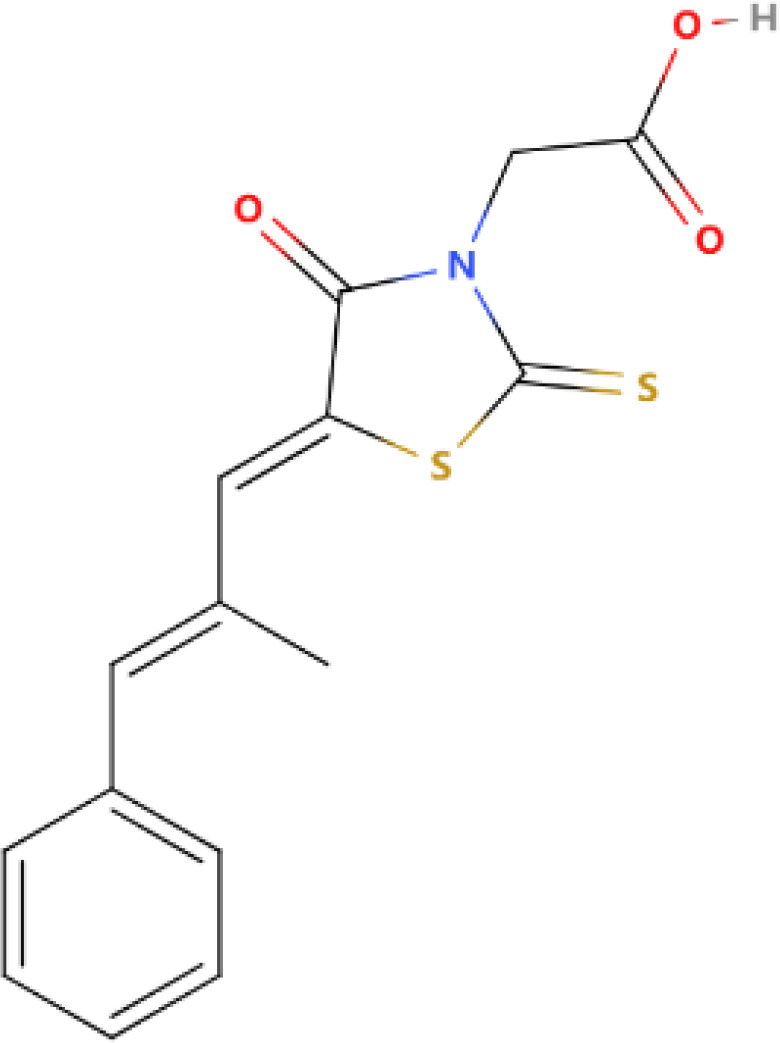	(11, 98)
HCCFA	0.0035	0.277	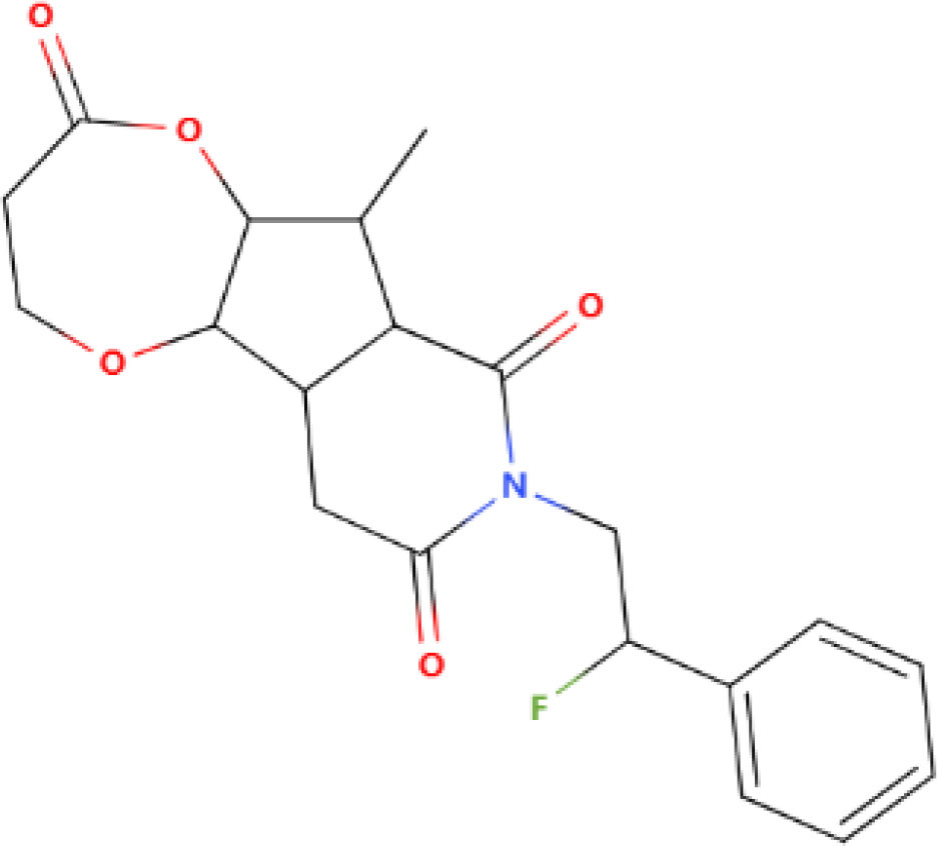	(11, 99)

## 5 Conclusions

**Figure 3:**
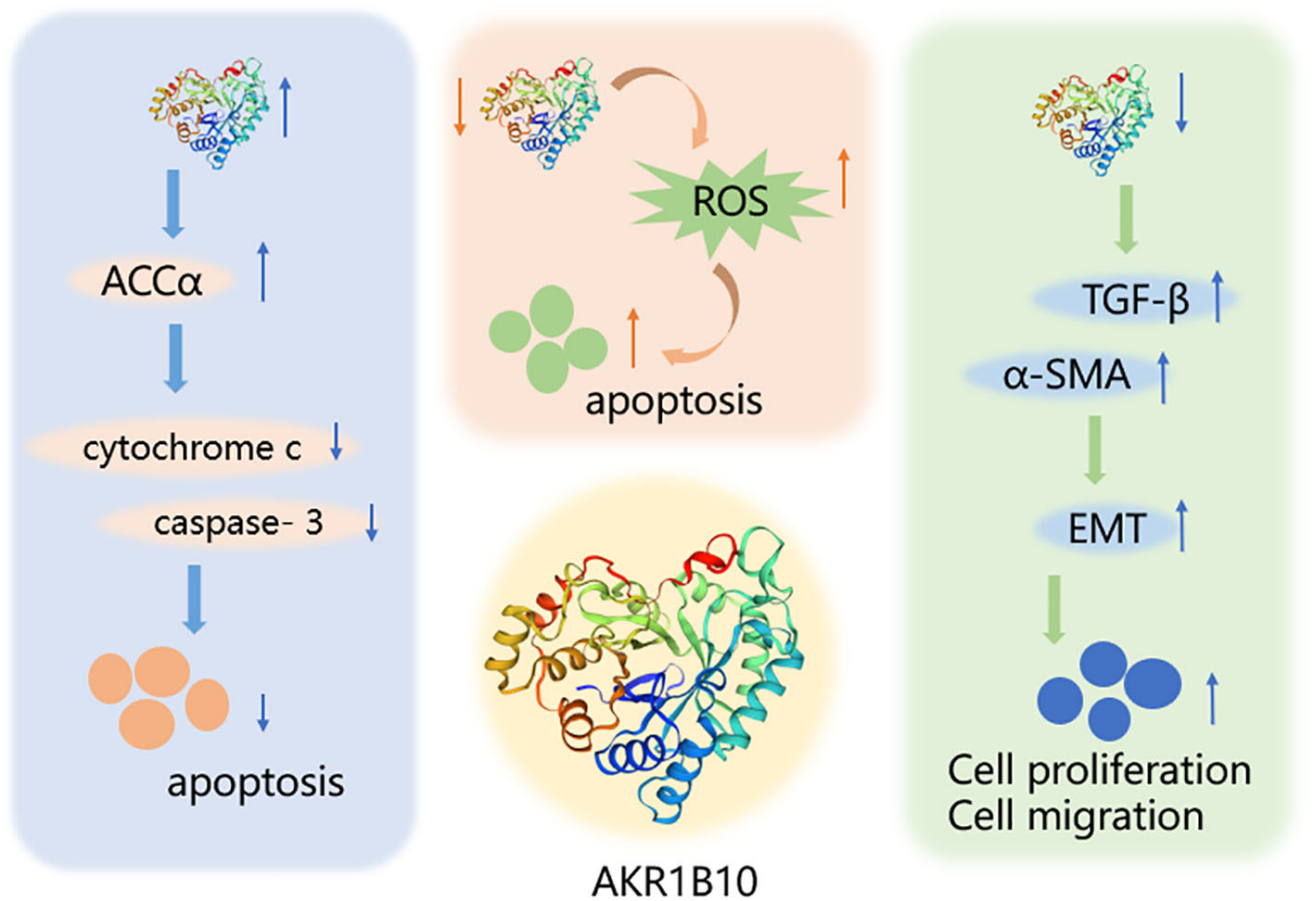
Mechanism of AKR1B10 in digestive system tumors. AKR1B10 can play a role in digestive system tumors by participating in lipid synthesis, oxidative stress, and epithelial mesenchymal transition. An upward arrow indicates an increase in content and a downward arrow indicates a decrease in content.

AKR1B10, one of the major members of the aldo-keto reductase family, has its gene located on chromosome 7q33 and consists of 316 amino acids, including a total of 13846 bases (100). AKR1B10 is involved in a variety of physiological activities, such as detoxification, and retinoic acid/retinol metabolism, and influences cell survival through the regulation of lipid synthesis, mitochondrial function and oxidative status, and carbonyl levels (101–103) (**Figure 3**). Since its discovery in 1998, AKR1B10 has been investigated as a potential biomarker in a number of oncological diseases, including breast, lung, endometrial, bladder, and renal cell carcinomas, as well as in a number of chronic diseases, such as alcoholic hepatitis, NAFLD, and benign prostatic hyperplasia (40, 104–110). This paper reviews the research progress on AKR1B10 in liver, gastric, colorectal, pancreatic, oral, laryngeal, and bile duct cancers, which are digestive tumor diseases, and emphasizes that, except for the downregulation of AKR1B10 expression in GC and CRC, AKR1B10 is overexpressed in the rest of the solid tumors, and it thus has potential as a diagnostic and prognostic indicator in all of these solid tumors.

Genome-Wide Association Studies (GWAS) studies have confirmed that HCC cells are significantly more dependent on AKR1B10, an enzyme that plays a crucial role in regulating the proliferation and migration of HCC cells. Importantly, AKR1B10 exhibits a high degree of specificity in HCC, which provides a theoretical basis for the development of specific inhibitors targeting AKR1B10, which are expected to improve patients’ quality of life by producing only minor toxicities in the treatment of HCC (111). In addition, data from large cohort studies further support the diagnostic value of AKR1B10 as a potential HCC serum marker superior to conventional AFP, which may contribute to the early diagnosis and prognostic assessment of HCC (112). Despite the remarkable progress of AKR1B10 in HCC, AKR1B10 and related GWAS studies in other GI tumors are still lacking. Given the multifunctionality of AKR1B10 in metabolism and tumor biology, exploring its expression pattern and function in more GI tumors will be a promising research direction.

**Figure 4:**
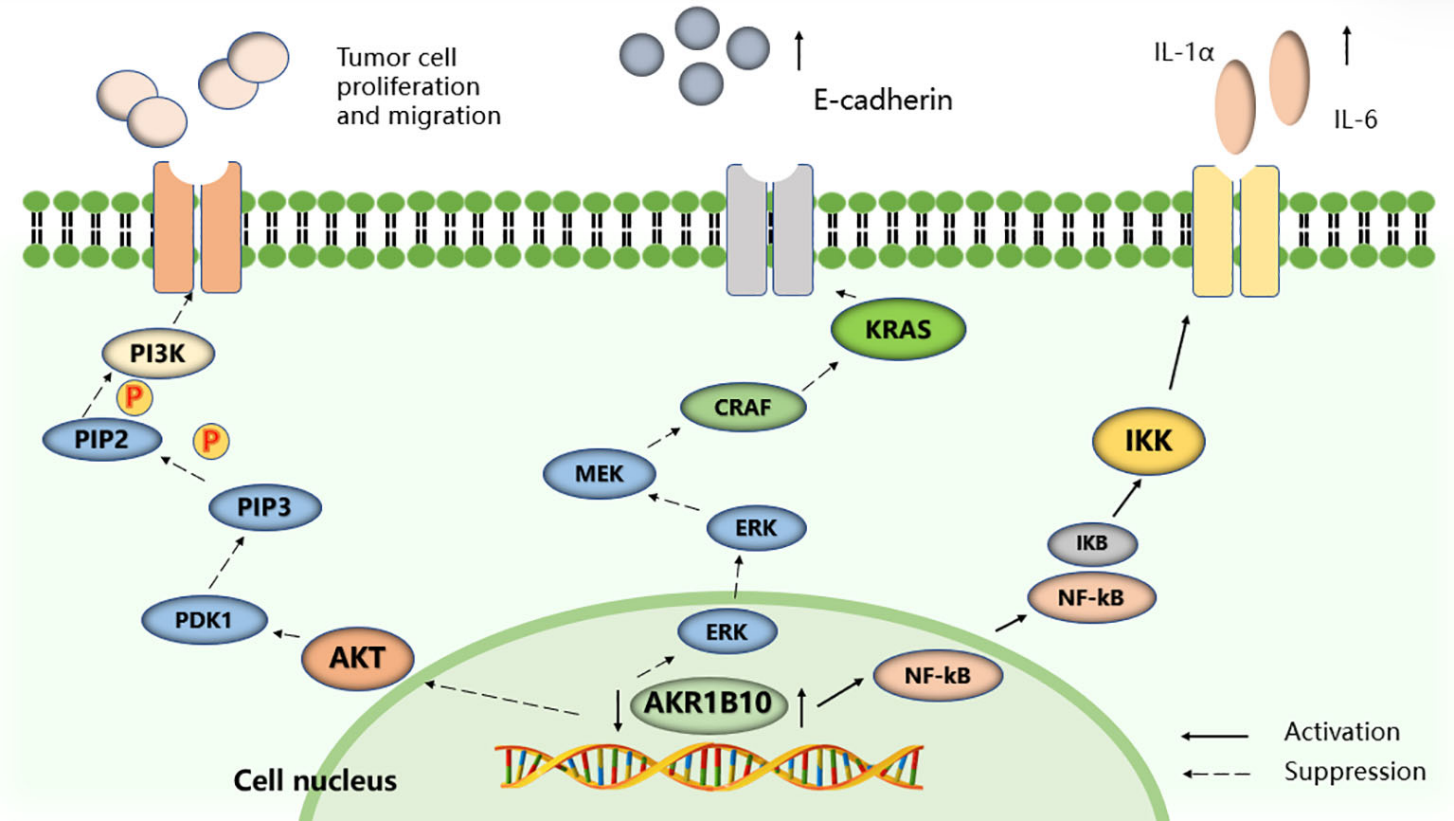
Selected signaling pathways regulated by AKR1B10. AKR1B10 promotes IL-1α and IL-6 production in colon cancer cells by activating the NF-KB signaling pathway. Silencing the expression of AKR1B10 can regulate the Kras-E-cadherin pathway and inhibit the proliferation, invasion and metastasis of pancreatic cancer cells, and reduce the phosphorylation level of PI3K and AKT to promote the proliferation, migration and invasion of HCC cells.

The study of AKR1B10 and its relationship with digestive system tumors has revealed its potential role in regulating the proliferation and migration of tumor cells through involvement in the PI3K/AKT and Kras signaling pathways. The development of highly selective AKR1B10 inhibitors offers promising prospects for improving tumor treatment and survival rates for patients with cancer (**Figure 4**).

It is important to note that tumors are heterogeneous (113), and the pathogenic and metabolic mechanisms of AKR1B10 can differ between different tumor types. The expression of AKR1B10 may also vary across different sites and stages of the same tumor. For example, its expression is high in early to mid-stage HCC, but decreases in advanced stages (114). In addition, AKR1B10 expression is downregulated in pre- CCA, but increases after progression (54). In GC, high expression of AKR1B10 tends to predict a poor prognosis for patients undergoing surgical treatment (7). In addition to the tumor itself affecting AKR1B10, external factors such as therapeutic means and therapeutic drugs, and the nutritional status of the patient may also have an effect on the differential expression of AKR1B10 between different tumors.

Meanwhile, AKR1B10 may play different roles at different stages of tumor development. In the early and proliferative stages of tumors, AKR1B10 may play a tumorigenic role by promoting cell growth and proliferation, which is consistent with our observation that inhibition of AKR1B10 expression resulted in cell cycle arrest and impaired cell proliferation (37). However, in the post hepatectomy setting, high expression of AKR1B10 may be associated with liver repair and regenerative capacity, which may explain the association with favorable prognosis observed in the meta-analysis (38). The complexity of the tumor microenvironment may also contribute to this apparent contradiction. AKR1B10 may play different roles within tumor cells and in the tumor microenvironment, and these roles may affect both tumor growth and patient prognosis. Moreover, patient populations differ in terms of tumor stage, treatment modality, and genetic background, and these factors may collectively influence the relationship between AKR1B10 expression and prognosis.

Metabolic studies in tumors have gained increasing attention from researchers, particularly in addressing key issues such as detection, traceability, and the impact of metabolites on tumor development. Further, there is a growing focus on identifying effective targets involved in metabolic pathways (115, 116).

In summary, the exploration of AKR1B10 expression in tumors, investigation of its interactions, and research of the molecular mechanisms of AKR1B10 are important research directions. Combining *in vivo* and *in vitro* studies with clinical samples will likely yield valuable results, which can significantly contribute to early tumor diagnosis, prognosis, and the development of novel therapeutic approaches in the future.
